# In Silico Exploration of Alternative Conformational States of VDAC

**DOI:** 10.3390/molecules28083309

**Published:** 2023-04-08

**Authors:** Carmen Mannella

**Affiliations:** Department of Physiology and Center for Biomedical Engineering and Technology, University of Maryland School of Medicine, Baltimore, MD 21201, USA; cmannella@som.umaryland.edu

**Keywords:** VDAC, β-barrels, ion channels, mitochondria

## Abstract

VDAC (Voltage-Dependent Anion-selective Channel) is the primary metabolite pore in the mitochondrial outer membrane (OM). Atomic structures of VDAC, consistent with its physiological “open” state, are β-barrels formed by 19 transmembrane (TM) β-strands and an N-terminal segment (*NTERM*) that folds inside the pore lumen. However, structures are lacking for VDAC’s partially “closed” states. To provide clues about possible VDAC conformers, we used the RoseTTAFold neural network to predict structures for human and fungal VDAC sequences modified to mimic removal from the pore wall or lumen of “cryptic” domains, i.e., segments buried in atomic models yet accessible to antibodies in OM-bound VDAC. Predicted in vacuo structures for full-length VDAC sequences are 19-strand β-barrels similar to atomic models, but with weaker H-bonding between TM strands and reduced interactions between *NTERM* and the pore wall. Excision of combinations of “cryptic” subregions yields β-barrels with smaller diameters, wide gaps between N- and C-terminal β-strands, and in some cases disruption of the β-sheet (associated with strained backbone H-bond registration). Tandem repeats of modified VDAC sequences also were explored, as was domain swapping in monomer constructs. Implications of the results for possible alternative conformational states of VDAC are discussed.

## 1. Introduction

VDAC (Voltage-Dependent Anion-selective Channel) is the primary metabolite pore in the mitochondrial outer membrane (OM) [1]. It occurs in the membrane at high packing density (10^3^–10^4^ per square micrometer) [2], with each “open” pore allowing rapid diffusion of ions (10^6^/msec) and larger metabolite molecules (10^2^–10^3^/msec) needed to support the chemiosmotic machinery inside mitochondria [3]. As its name implies, VDAC exhibits multiple conductance states in bilayers that are gated by transmembrane voltage and by interactions with cytoplasmic proteins, such as hexokinase and tubulin dimers [4,5,6]. While the lower conductance, cation-selective states of VDAC are still very permeable to small monovalent anions and cations (such as Na^+^, K^+^, and Cl^−^), they are almost impermeable to important metabolites, such as adenine nucleotides [7,8], and are tenfold more permeable to the divalent cation Ca^2+^, which regulates several mitochondrial metabolic pathways [9]. Thus, VDAC can be considered a cellular “hub” or “gateway” that strongly influences energy metabolism and mitochondrially localized signaling pathways, such as apoptosis [10,11], all of which perform important roles in human diseases, such as cardiac disorders, Parkinson’s and cancer [12,13,14].

**Figure 1:**
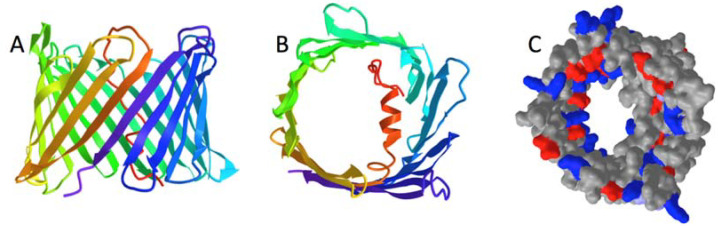
Atomic models for human VDAC1 (hVDAC1) from (**A**,**B**) NMR and (**C**) X-ray crystallography. Side (**A**) and top-down (**B**) views of the polypeptide chain of PDB 2K4T [15]. For these and subsequent ribbon models, mitochondrial intermembrane space (IMS) is down and cytosol up in side views; top views correspond to 90° rotation towards the viewer. The spectrum color scheme used is RED at N-terminus and VIOLET at C-terminus. (**C**) Space-filling (van der Waals) model for top-down view of the polypeptide chain of PDB 2JK4 [16] with cationic residues BLUE and anionic residues RED, illustrating how the inserted N-terminal domain narrows the pore to a projected size of approximately 12 × 15 Å. A molecule of ATP (MW 563), roughly a rod 15 Å long and 5–8 Å across, can diffuses at a rate of ~700 molecules/msec through this “open” VDAC pore, but cannot diffuse through the voltage-gated “closed” pore (structure unknown) [7,8].

Beginning in 2008, atomic-resolution structures were obtained for bacterially expressed VDACs in various lipid or detergent systems by NMR and X-ray crystallography [15,16,17]. In derived atomic models (one of which is shown in Figure 1), the pore is formed by a β-barrel (cylindrically curved antiparallel β-sheet) containing 19 transmembrane (TM) β-strands. The strands are predominantly amphipathic, with polar residues facing inside the pore lumen and non-polar facing outward, and the cylindrical structure is “sealed” by H-bonding between the two parallel β-strands at the N- and C-terminal regions, i.e., β1 and β19. The N-terminal segment (*NTERM*) of 20–40 residues (isoform dependent) contains a short α-helical region and folds inside the lumen where it interacts with inward-facing residues of the β-strands, mainly in the C-terminal half of the β-barrel (i.e., strands β9–19).

Atomic models generated by NMR or X-ray crystallography are generally consistent with the electrophysiological properties of the bilayer “open” state and with the projected density maps of OM-bound VDAC pores obtained by cryo-EM [18]. Thus, they are used as the base structure for computational studies of VDAC’s permeability properties, gating and protein interactions [19,20,21,22]. Changes in VDAC permeability to metabolites (like ATP, see legend of Figure 1) associated with partially “closed” states have been generally attributed to rearrangements of the N-terminal segment and/or distortions in shape of the β-barrel pore, e.g., [19,23]. However, the structures of the lower permeability substates of VDAC have not yet been obtained experimentally.

There is considerable evidence (pre-2008) that several VDAC domains buried in atomic models are accessible to topological probes (such as sequence-specific antibodies and proteases) in mitochondria and isolated outer membranes, summarized in ref. [3]. These “cryptic” domains include *NTERM* in fungal and mammalian VDAC and, in fungal VDAC, the C-terminal β-strand (β19) and an internal β-hairpin β13–14 [24,25,26]. Fab directed against *NTERM* maps to regions between pores in ordered OM arrays of fungal VDAC, suggesting this domain extends away from the lumen in at least some of the pores [27]. These results were generally consistent with conclusions drawn from electrophysiological studies of yeast VDAC reconstituted in lipid bilayers. “Open” VDAC has several domains (including β13–14) not in the ion conduction pathway, but later shown to be part of the β-barrel pore, and voltage-induced closure is associated with removal from the pore of additional domains at the N- and C-termini [8,28]. The involvement of reversible large-scale conformational changes in VDAC closure also was inferred from significant decreases in both pore volume [29] and β-sheet content [30] associated with voltage- and pH-induced gating, respectively.

We have proposed that in the mitochondrial outer membrane VDAC occurs as an ensemble of conformers that vary in their permeability properties and interactions with proteins in the outer membrane, intermembrane space, and cytosol, including itself [3]. The aim of the current study is to explore possible alternative conformational states of VDAC inferred from the above described topological and biophysical data. The approach uses a neural network to simulate folding of VDAC polypeptides lacking the “cryptic” (accessible) domains, to mimic their removal from the pore wall or interior. The approach is agnostic about what might trigger domain movement, and instead asks how it affects the intrinsic structure of the β-barrel pore in vacuo, i.e., not inserted in a lipid bilayer. As will be explained, predictions for full-length VDAC sequences are similar to the β-barrel motif of atomic models, but with a weaker backbone H-bonding, and the topologies predicted for modified sequences display distortions that likely reflect strain in the backbone β-sheet resulting from domain excision [31,32,33]. Implications for predicting alternative VDAC conformations are discussed.

## 2. Results

### 2.1. Folding Predictions for VDAC Monomer Constructs

In the last two years, considerable attention has been paid to so-called “artificial intelligence” approaches for predicting protein folding, and the increasing accessibility of algorithms, such as AlphaFold2 [34] and RoseTTAFold [35] for various applications [36]. We decided to use the three-track neural network RoseTTAFold [35] to predict possible structures for VDAC sequences modified to mimic removal of experimentally determined accessible segments (“cryptic” domains) from the pore wall or interior. This was completed for human VDAC isoforms hVDAC1 and hVDAC2, and for *N. crassa* VDAC (ncVDAC). Folding motifs predicted by RoseTTAFold for full-length and truncated sequences of hVDAC1, hVDAC2, and ncVDAC are summarized in Table 1.

The predicted topologies fall broadly into five classes:

- β-barrels with varying gaps (4.6–17.5 Å) between N- and C-terminal β-strands (“N-C gap”, orthogonal distance between adjacent β-strands measured at Cα atoms).
- “Partial” β-barrels with very wide “N-C gap” (28–31 Å) measured normal to cylinder axis.
- “Disrupted” β-barrels with short segments containing 4–6 β-strands tilted away from the main β-sheet having 9–12 β-strands.
- “Reverse” β-barrels, with left-hand rather than the normal right-hand twist observed in anti-parallel β-sheets in proteins.
- Collapsed β-sheet structures with no pore.

#### 2.1.1. Predicted Topologies for hVDAC1

**Figure 2:**
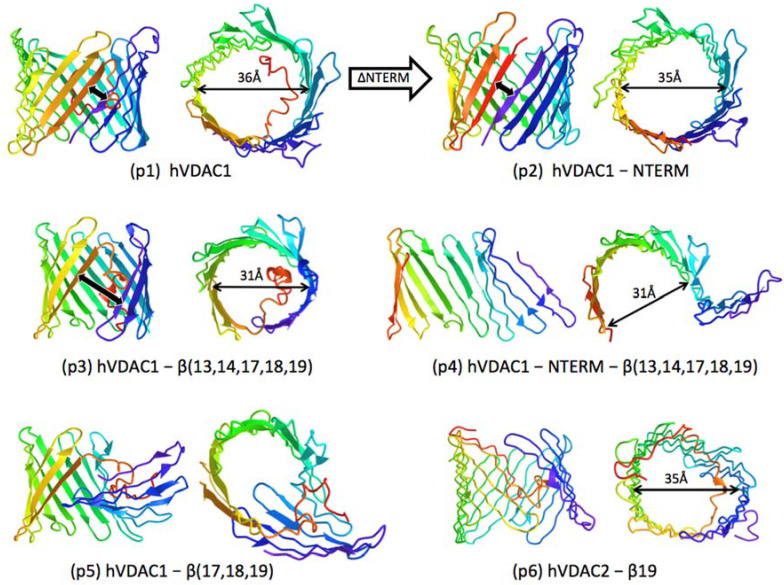
Examples of predicted topologies for VDAC constructs corresponding to *Class I* (p1, p2, and p3), *Class II* (p4), *Class III* (p5), *Class IV* (p6), as explained in text. Dimensions indicated are measured normal to the cylinder axis at the Cα backbone near the middle of each β-barrel.

The prediction for full-length hVDAC1, (p1) in Figure 2, is a *Class I* β-barrel with 19 transmembrane [TM] strands within +/−2 residues of those in the atomic models and similar lumen dimensions, e.g., 36 Å at the Cα backbone in the direction indicated in (p1). However, H-bonds between 8 of the 19 TM strands are stretched >3.8 Å, indicated by absence of flat β-strand arrows in the ribbon diagrams. The N-C gap (thick black arrow, is 4.7 Å, as in atomic models, and the parallel terminal β-strands (β1 and β19) are H-bonded. The N-terminal segment (*NTERM*) makes 33 contacts (largely van der Waals) with residues in β-strands β7–19, about half as many as in atomic models.

The prediction for truncation of *NTERM* (p2) is a *Class I* β-barrel with minor shifting in H-bond registration, more H-bond stretching (involving 11 of 19 TM strands) and more vertical tilt to TM strands, e.g., β1 is tilted 40° relative to the cylinder axis in (p1) and 30° in (p2). The horizontal pore dimension (35 Å) and N-C gap (4.6 Å, H-bonded) are similar to that for the unmodified hVDAC1 sequence (p1). Excision from hVDAC1 of β-strand segments corresponding to β-hairpin β13–14, alone or in combination with successive removal of C-terminal strands β19 to β17, usually yields *Class I* β-barrels even with only 14 β-strands (p3). The same 14-strand VDAC construct without *NTERM* yields *Class II* topology (p4), a “partial” β-barrel with two domains, a curved β-sheet consisting of β1–9 and a short flat β-sheet “flap”, comprised of the remaining five TM strands, that swings away from the β-barrel. The diameter of the β-cylinder in (p3) and curved β-sheet in (p4) are the same (31 Å), suggesting the β1−β9 domain performs an important role in establishing curvature of the VDAC pore.

In general, predictions for hVDAC1 constructs containing *NTERM* have that domain inside the β-barrel with one exception: excision of the three C-terminal TM strands β17–19 yields a *Class III* fold, (p5) in Figure 2. This topology is similar to *Class II*, consisting of the β1–9 curved β-sheet and a shorter, flat β-sheet formed by the remaining β-strands. However, instead of swinging away from the β-barrel as in *Class II*, the short “flap” tilts roughly normal to the cylinder axis, held in place by interactions with *NTERM*, visible as the extended red random coil in (p5).

**Figure 3:**
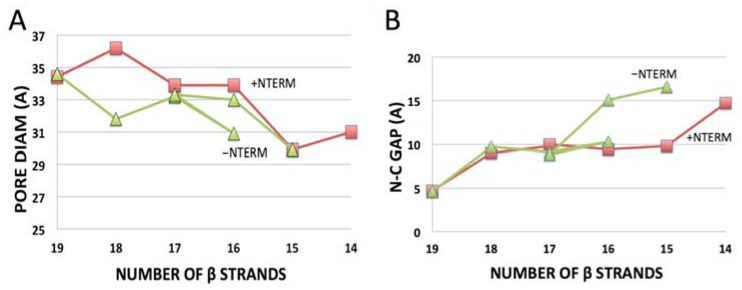
Systematic variation in (**A**) pore diameter and (**B**) “N-C gap” between β-strands at the N- and C-termini of *Class I* topologies with extent of domain truncation in hVDAC1 sequences. (**A**) Diameters measured as average of largest and smallest dimensions normal to the cylinder axis at the Cα backbone near the middle of each β-barrel. (**B**) N-C gaps measured as described in Section 2.1 (data from Table 1).

As noted above, the majority of predictions for modified hVDAC1 sequences (15 of 17) are Class I β-barrel topologies with generally smaller diameters than the atomic model (34–30 Å, see Figure 3A), and roughly circular profiles (ellipticities in the range 0.8 to 1.0, compared to 0.9 for the atomic model). The “N-C gap” increases systematically as the number of TM strands decreases (Table 1 and Figure 3B), suggesting it is an indicator of intrinsic strain in the β-sheet backbone caused by strand truncation. This strain might be sufficiently compensated by the free energy gain of membrane insertion [37] for the β-barrel to compress and close N-C gaps (by H-bonding), resulting in stable, but smaller OM pores. In contrast, deformations in the *Class II and III* folds (larger N-C gaps and disruptions in β-sheet backbone) suggest much larger strain in the β-sheet H-bond registration, which the neural network did not tend to radically adjust.

#### 2.1.2. Predicted Topologies for hVDAC2 and ncVDAC

A different pattern emerges (Table 1) with hVDAC2 and ncVDAC which, unlike hVDAC1, lack determined atomic structures in the protein database. [Overall homology between hVDAC1 and hVDAC2 is 68%, but the latter has a significantly longer N-terminal segment. The protein database contains a structure for zebrafish VDAC2, which also lacks the longer N-terminal segment of hVDAC2 [38]. A pig VDAC2 model in the database is a predicted fit to a 35 Å density map of a mitochondrial OM protein complex from cryo-EM, with considerable uncertainty in the pore region [39]]. Both full-length hVDAC2 and ncVDAC sequences yield β-barrels with 19 TM strands that closely match the predicted secondary structure in the literature, e.g., [40,41]. However, N-C gaps for both full-length sequences are too large (>7 Å) for H-bonding to occur between β1 and β19. Either these β-barrels are inherently more strained than hVDAC1 or lack of prior 3D information affects the quality of predictions. There are several indications that the latter may be the case, in particular, missing information about interactions of *NTERM* with the β-barrel: (1) Truncation of *NTERM* narrows N-C gaps for otherwise unmodified hVDAC2 and ncVDAC, not the case for hVDAC1 (Table 1), suggesting NTERM interactions with the β-barrels creates backbone strain. (2) *Class I* β-barrels are predicted for 11 of 15 modified hVDAC2 and ncVDAC constructs *without NTERM,* but only for 4 of 15 *with NTERM*. This outcome contrasts sharply with that obtained with hVDAC1, for which 13 of 15 predictions for modified constructs are *Class I* β-barrels and presence of *NTERM* is associated with the same or smaller N-C gaps (lower distortion) than occur when *NTERM* is removed (Table 1 and Figure 3B).

In the case of hVDAC2, about half of the predictions for modified sequences (7 of 15) are *Class II* and *III*, characterized by discontinuities in the β-sheet backbone. Predictions for ncVDAC are more diverse, with 3 of 15 in *Class II* and *III* and another 3 having an even more unusual fold, also predicted for one hVDAC2 construct. These are *Class IV* topologies, (p6) in Figure 2, consisting of a cylindrical anti-parallel β-sheet with left-hand twist, instead of the right-hand twist exclusively seen in nature [42]. The reverse twist is considered energetically disfavored and involves large-scale systematic shifts in H-bond registration in β-sheets [42]. In fact, most H-bonds are weak (stretched) in the *Class IV* predictions, attested by absence of flat β-strand arrows in the ribbon diagram of (p6), although orientation of residues (polar facing inside the β-barrel and nonpolar facing outward) is retained. Finally, two of the ncVDAC predictions fall in *Class V*, a collapsed structure with no extended curved β-sheet.

### 2.2. Predictions for VDAC Dimers and Domain Swapping

**Figure 4:**
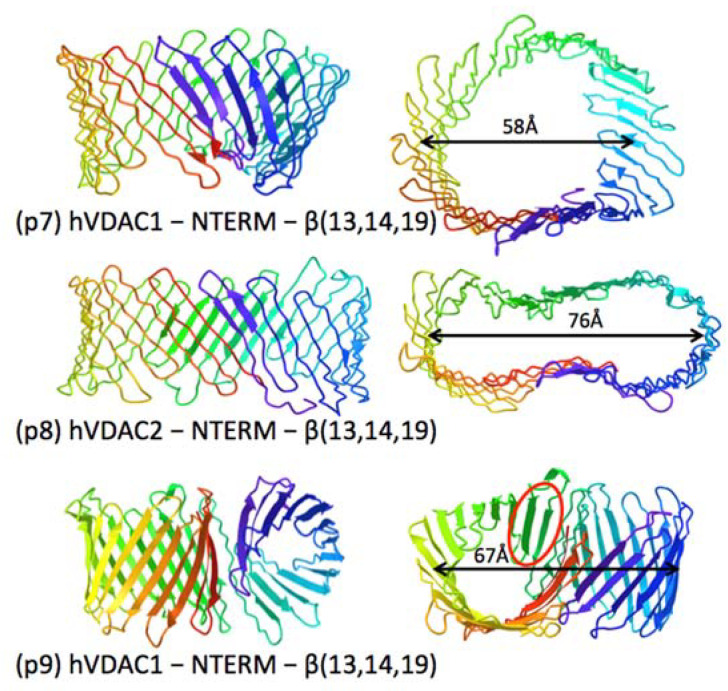
Folding predictions for hVDAC tandem repeat constructs.

Folding simulations were run using sequence constructs consisting of tandem repeats of full-length or modified VDAC sequences. Predictions almost always consisted of two separately folded β-sheet domains similar to those observed with monomer sequences. However, this was not always the case with human VDAC sequences modified to remove the three “cryptic” domains *NTERM*, internal β-hairpin β13–14, and C-terminal β-strand β19. The hVDAC1 and hVDAC2 monomer constructs fold as 16-strand *Class I* β-barrels with N-C gaps of ~10 Å (Table 1). When run as tandem repeats in RoseTTAFold, the tandem repeat constructs yielded (in some runs) single large 32-strand β-barrels, (p7) and (p8) in Figure 4, with reverse handedness as in *Class IV* monomers. The hVDAC1 “dimer pore” is roughly circular with an average diameter of ~60 Å, while that for hVDAC2 is elongated and bilobed with linear dimension of 76 Å. In both cases, a flat antiparallel β-sheet consisting of 4 or 5 β-strands (with unstretched H-bonds) at the C-terminus of one monomer appears to set the H-bonding register for the entire structure, which retains the amphipathic orientation of polar residues facing inside the lumen and non-polar facing out. 

In a few tandem repeat runs, folding motifs were obtained in which β-strands from one β-sheet were inserted between the N- and C-terminal β-strands of a β-barrel formed by the other β-sheet. An example is shown in (p9) of Figure 4 for the same 16-stranded hVDAC1 construct that produced the dimer β-barrel of (p7). The predicted fold has a β-barrel on the left with 18 β-strands, with the two strands circled in red corresponding to the β1–2 hairpin from second β-sheet, which has inserted between β1 and β16 of the first β-barrel.

In other folding simulations, domains consisting of one or two β-strands were systematically inserted into the hVDAC1 sequence at various locations with or without *NTERM*. In general, the inserted domains *did not* integrate into the backbone β-barrel motif, and instead folded outside the pore as a random coil or in a few cases, including the β13–14 hairpin, as an α-helix. Exceptions to this behavior occurred at the interface between β1 and β19, where insertion of an additional β1–2 domain at the N-terminus (after deletion of the *NTERM* domain) yielded an undistorted 21-strand β-barrel with H-bonded N-C gap.

## 3. Discussion

This novel application of the RoseTTAFold neural network provides a survey of possible alternative conformational states of the mitochondrial pore VDAC. The approach has advantages of speed and flexibility, with most folding simulations taking only a few minutes to run on resources provided by the Web-based RoseTTAFold.ipynb portal. The resulting predictions are in no way a substitute for the rigorous computational and experimental approaches required to evaluate the energetics of various structural models of this gated pore. Rather the approach is intended to stimulate thinking about the nature of VDAC conformers and possibly guide efforts to determine the actual structures of “closed” states of VDAC.

### 3.1. Support for Basic Premise

The simulations suggest that removal of short “cryptic” domains creates “backbone strain” that, when severe, frustrates normal protein folding, as has been suggested for soluble β-sheet proteins [32,33]. The severity of distortions predicted for VDAC folding vary from widening of the distance between β-strands at the N- and C-termini, to breaks in the core β-sheet, to reversal in twist of the β-sheet (which involves global shift in registration of β-strand H-bonding). We propose that the strain associated with minor distortions (i.e., small N-C gaps) might be compensated in native VDAC by the energy of stabilization from membrane insertion [37]. This is supported by experimental and computational studies of VDACs engineered with similar modifications:

- Deletion of the C-terminal β-strand from the sequence of ncVDAC yields a *Class I* β-barrel with or without (+/−) *NTERM*, with about equal N-C gaps (9.4 vs. 9.8 Å) (Table 1). The actual engineered construct yields a functional, voltage-gated pore with slightly smaller conductance than the wild-type 19-strand β-barrel in bilayers [40], consistent with the premise that *Class I* topologies with small N-C gaps can fold as functional β-barrels in a membrane environment.
- hVDAC2 constructs engineered with deletions of one to three β-strands at the C-terminal show pore-forming activity in bilayers, although only the 17-stranded variant display voltage-gating similar to the 19-stranded wild-type [41]. All three truncated sequences yield *Class I* folding predictions for hVDAC2 without *NTERM* (Table 1), but only the 17-stranded and wildtype 19-stranded sequences (corresponding to gatable pores) yield *Class I* topologies with *NTERM* present. The results are again *consistent with the premise that Class I predictions represent pores in membranes*, although the inverse is not necessarily true when *NTERM* is present. Instead, interactions (entanglement) between an extended N-terminal domain and the β-sheet appear to frustrate folding of the polypeptide into *Class I* β-barrels (evident in (p5) Figure 2) for truncation of β19–17 with both hVDAC2 and hVDAC1.

### 3.2. Structural Role of the N-Terminal Domain

There is considerable structural and functional evidence that the N-terminal segment of VDAC stabilizes the 19-strand β-barrel and might influence both the shape of the pore (circular or elliptical) and its ability to oligomerize, e.g., [19,23,40,43,44,45]. RoseTTAFold predictions for hVDAC1 are generally consistent with a structural role for *NTERM*, e.g., presence of *NTERM* correlates with wider (more open) hVDAC1 pores than with the *NTERM* removed (Figure 3A). In the most extreme case (excision of 5 β-strands), the 14 β-strand hVDAC1 construct folds as a *Class I* β-barrel pore with *NTERM* present but yields a *Class II* “partial pore” without *NTERM*, (p3) and (p4), respectively (Figure 2). Interactions of a tightly folded, partly helical *NTERM* domain with the β-barrel in (p3) appear to stabilize the pore structure despite its large (17.5 Å) N-C gap.

However, the same is not true for hVDAC2 and ncVDAC for which, as already noted, the presence of *NTERM* correlates with increased frequency of deformed *Class III–V* topologies (Table 1). Even unmodified, full-length hVDAC2 and ncVDAC sequences yield *Class I* β-barrels with wider N-C gaps (7.3, 7.5 Å) than predicted for unmodified hVDAC1 (4.7 Å), suggesting greater intrinsic backbone strain in predicted structures for the former *that is reduced* (smaller N-C gaps) by removal of *NTERM*. Why the N-terminal domain would destabilize hVDAC2 and ncVDAC, but have an opposite, stabilizing effect on hVDAC1 is unclear. It has been suggested (based on the previously cited work on hVDAC2 variants [41]) that the 19-strand β-barrel has been evolutionarily selected for *because it is inherently metastable* and thus more readily gated. However, this argument applies to all VDACs, while there are clearly differences between predictions for hVDAC1 and hVDAC2/ncVDAC. In this context, it is worth recalling that the protein database contains several atomic resolution structures for mammalian VDAC1, but none for mammalian VDAC2 or fungal VDAC. Thus, it seems possible (perhaps likely) that absence of 3D structural information about the latter proteins may negatively impact the ability of RoseTTAFold to predict the interactions between the N-terminal domain and β-barrel in these cases.

### 3.3. Implications of the RoseTTAFold Predictions for VDAC Conformers

Overall, these results imply that there is sufficient flexibility in the β-barrel backbone of VDAC to accommodate removal of multiple β-strands suggested by earlier topological studies. If we compare RoseTTAFold predictions only for *-NTERM* constructs (due to apparent uncertainties about *NTERM* interactions for hVDAC2 and ncVDAC) we see consensus *Class I* folds (likely functional pores) with all three VDACs for removal of β19 alone or together with β18, and for β19 along with the internal β13–14 hairpin. The latter combination is the minimum subset of “cryptic” β-strands derived from the topological data. It could be argued (based on considerations in Section 3.2) that movement of the N-terminal domain out of the pore before the β-strands would help avoid the entanglements that underly most Class III (disrupted β-sheet) predictions. However, hVDAC1 results indicate that presence of *NTERM* inside the pore helps to stabilize β-barrels with smaller numbers of β-strands, e.g., (p3) in Figure 2 and Figure 3B. This is consistent with evidence that *NTERM* stays attached to β11 during voltage gating [46], so either *NTERM* remains at least partially in the pore to stabilize the voltage-closed conformer, or motion of *NTERM* and certain β-strands during closure is coordinated.

### 3.4. Implications for VDAC-VDAC Interactions

Tandem repeat constructs were used in folding simulations to look for indications of preferred interactions between VDAC molecules. For example, recent studies have shown preferred dimer interactions between VDAC proteins from zebrafish [38] and oligomers of VDAC pores have been implicated in macromolecular transport across the OM [44,47]. These larger VDAC pores are generally depicted as hexamers of the basic VDAC β-barrel arranged as observed in ordered arrays in the fungal OM [27,44].

The topologies predicted for tandem repeat constructs of human VDACs (Figure 4) are suggestive of a different kind of “megapore”, in which β-sheets from two VDAC polypeptides anneal end-to-end to form a single dimer pore. This “minority” outcome has been seen in only a few simulations run with the 16-strand hVDAC constructs with *NTERM*, β19 and the internal β13–14 hairpin excised. Superficially, the transition (p8) → (p7) suggests a possible sequence in formation of a “megapore”: alignment of two β-barrels at their N-C interfaces to create a bilobed “double-barrel” pore, followed by major changes in β-sheet curvature to form a single wide pore. A third “minority” outcome for folding of this tandem repeat, (p9) in Figure 4, is suggestive of a different process. In this predicted fold, the β1–2 hairpin from the second β-sheet in the construct inserts between strands β1 and β19 of the β-barrel of the first VDAC sequence in the tandem repeat. This replicates the initial step in a model for sorting β-barrel precursors through the “lateral gate” in SAM50, a β-barrel protein that inserts other β-barrel proteins into the OM [48]. In the model, the first β-hairpin insertion step is proposed to be followed by progressive insertion of the remaining β-strands of the precursor, yielding an intermediate state, such as (p7), and finally release of the complete new β-barrel, which at some time point might look like the bilobed structure in (p8). [Note that creating two smaller β-barrels from one wide pore would likely involve wide-ranging shifts in registration of H-bonding. The reversal of β-sheet twist in predicted folds (p7) and (p8) might reflect failure of the neural network to establish proper “biological” H-bond registration for extreme topologies not represented in the protein database.] While these folding simulations with tandem repeat VDAC constructs occur at low frequency, they might illustrate types of molecular maneuvering common to β-barrel interactions. For example, there is direct evidence that VDAC catalyzes its own insertion into membrane bilayers [49]. Based on these observations, we speculate that both insertion and closure of VDAC might involve sliding β-hairpins (induced by interactions of VDAC with other proteins, including itself), and that postulated VDAC “megapores” might represent an intermediate stage (p7) in the insertion process.

## 4. Materials and Methods

We used the three-track neural network described by Baek et al. [35] to predict possible structures for VDAC sequences modified to mimic removal of experimentally determined accessible segments (“cryptic” domains) from the pore wall or interior. This was completed for human VDAC isoforms hVDAC1 and hVDAC2, and VDAC from *N. crassa* (ncVDAC). The neural network operates by “successive transformation and integration” of information from 1D sequences, 2D distances, and 3D coordinates [23]. Our implementation employed the Web-based RoseTTAFold.ipynb portal provided by David Baker’s lab (Univ of Washington) through Google-Colab in interactive mode. *mmseqs2* (FAST method from ColabFold) was used for multisequence alignment, *RoseTTAFold* for mainchain prediction and *Scrwl4* for sidechain prediction.

Predictions for unmodified sequences of hVDAC1, hVDAC2, and ncVDAC yielded 19-strand β-barrels similar to the experimentally determined atomic model with good confidence based on overall Local Distance Difference Test [50], lDDT = 0.81, 0.81, and 0.77, respectively. Truncated sequences generally yielded one or two likely predictions (lDDT = 0.88–0.76) that usually differed at regions of low confidence (lDDT < 0.60). VDAC dimer constructs were simulated by tandem repeat of full-length or modified VDAC sequences, sometimes including short connector regions. Predictions for dimer constructs typically had lower overall confidence (lDDT = 0.72–0.67) and considerably more variability than those for monomers.

All folding models shown were generated from the pred.pdb output files of RoseTTAFold using the NCBI Web-based 3D structure viewer iCn3D [51,52]. The tools in iCn3D were used to measure three-dimensional distances in the models, and characterize intramolecular interactions, such as backbone H-bonding and van der Waals contacts between N-terminal and β-sheet residues.

## 5. Conclusions

This novel application of RoseTTAFold to an integral membrane β-barrel protein, VDAC, provides useful information about both the protein and the neural network. Despite absence of a membrane environment, RoseTTAFold reproduces the fundamental topology of human VDAC isoform 1 (hVDAC1), which has multiple atomic structures in the protein database. Deletion of specific domains (implicated as mobile by earlier studies) generally yields β-barrels with minor distortions, namely, gaps of 10–15 Å between N- and C-terminal β-strands (“N-C gaps”). Similar β-barrel topologies are obtained with two other VDAC molecules (hVDAC2 and ncVDAC) *not represented* in the protein database, but only after excision of the short (20–40 residue) N-terminal domain, which otherwise gets entangled in the β-strands and loops of variant pores. We suspect this is due to the absence of explicit 3D information about the latter two VDACs. Since several of the VDAC variants have been shown experimentally to yield functional pores, the small N-C gaps in predicted folds likely are indicators of intrinsic strain in the β-barrel that is compensated by the energy of stabilization of membrane insertion. Folding simulations run with tandem repeats of VDAC sequences illustrate possible modes of interaction between VDAC molecules that may be relevant to processes, such as membrane insertion, pore closure and formation of “megapores”.

## Figures and Tables

**Table 1:** Folding predictions for VDAC monomer constructs ^1^.

CONSTRUCT + NTERM	CLASS	N-C GAP (Å)	lDDT	CONSTRUCT − NTERM	CLASS	N-C GAP (Å)	lDDT
HVDAC1	I	4.7	0.81	HVDAC1 - N	I	4.6	0.88
HVDAC1 - β19	I	9.0	0.81	HVDAC1 - N,β19	I	9.7	0.88
HVDAC1 - β(18–19)	I	9.8	0.81	HVDAC1 - N,β(18–19)	I	9.1	0.89
HVDAC1 - β(17–19)	III	−	0.81	HVDAC1 - N,β(17–19)	I	10.3	0.89
HVDAC1 - β(13–14)	I	10.0	0.80	HVDAC1 - N,β(13–14)	I	8.8	0.85
HVDAC1 - β(13–14,19)	I	9.4	0.80	HVDAC1 - N,β(13–14,19)	I	15.1	0.86
HVDAC1 - β(13–14,18–19)	I	9.8	0.80	HVDAC1 - N,β(13–14,18–19)	I	16.6	0.85
HVDAC1 - β(13–14,17–19)	I	14.7	0.78	HVDAC1 - N,β(13–14,17–19)	II	31.0	0.88
HVDAC2	I	7.5	0.81	HVDAC2 - N	I	5.3	0.88
HVDAC2 - β19	IV	−	0.78	HVDAC2 - N,β19	I	9.9	0.85
HVDAC2 - β(18–19)	I	9.7	0.78	HVDAC2 - N,β(18,19)	I	9.1	0.88
HVDAC2 - β(17–19)	III	−	0.80	HVDAC2 - N,β(17–19)	I	16.9	0.87
HVDAC2 - β(13–14)	III	−	0.76	HVDAC2 - N,β(13–14)	III	−	0.82
HVDAC2 - β(13–14,19)	III	−	0.76	HVDAC2 - N,β(13–14,19)	I	9.7	0.83
HVDAC2 - β(13–14,18–19)	III	−	0.76	HVDAC2 - N,β(13–14,18–19)	II	28.5	0.85
HVDAC2 - β(13–14,17–19)	I	17.5	0.77	HVDAC2 - N,β(13–14,17–19)	II	29.7	0.86
NCVDAC	I	7.3	0.77	NCVDAC - N	I	5.6	0.84
NCVDAC - β19	I	9.8	0.79	NCVDAC - N,β19	I	9.4	0.83
NCVDAC - β(18–19)	V	−	0.78	NCVDAC - N,β(18–19)	I	9.4	0.85
NCVDAC - β(17–19)	III, IV	−	0.77	NCVDAC - N,β(17–19)	II	27.9	0.87
NCVDAC - β(13–14)	IV	−	0.77	NCVDAC - N,β(13–14)	IV	−	0.84
NCVDAC - β(13–14,19)	V	−	0.76	NCVDAC - N,β(13–14,19)	I	10.4	0.84
NCVDAC - β(13–14,18–19)	I	10.6	0.78	NCVDAC - N,β(13–14,18–19)	I	10.6	0.85
NCVDAC - β(13–14,17–19)	III	−	0.77	NCVDAC - N,β(13–14,17–19)	I	10.0	0.84

^1^ Results for systematic deletion of the internal β-hairpin (β13–14) and one to three β-strands at the C-terminus (β19,18,17) are shown for constructs with (left) and without (right) the N-terminal segment (+/− NTERM). Note that “N-C gap” (defined in text) is measured between backbone Cα atoms, unlike H-bonds, which occur between backbone Cα and N atoms. IDDT is a measure of confidence in the overall prediction with values > 90 very high confidence and < 50 very low confidence (see Section 4). Each construct was used for multiple folding simulations (at least three) with the results in the table corresponding to the most frequent prediction.

## Data Availability

A file containing the RoseTTAFold constructs and outputs for this study will be made available upon request to the author.
